# Study of the Wavelength Dependence in Laser Ablation of Advanced Ceramics and Glass-Ceramic Materials in the Nanosecond Range

**DOI:** 10.3390/ma6115302

**Published:** 2013-11-19

**Authors:** Daniel Sola, Jose I. Peña

**Affiliations:** Department of Science and Technology of Materials and Fluids, Material Science Institute of Aragon, University of Zaragoza-Spanish National Research Council (CSIC), Maria de Luna 3, Zaragoza 50.018, Spain; E-Mail: jipena@unizar.es

**Keywords:** laser ablation, glass-ceramic, advanced ceramics, reference position, hardness

## Abstract

In this work, geometrical dimensions and ablation yields as a function of the machining method and reference position were studied when advanced ceramics and glass-ceramic materials were machined with pulsed lasers in the nanosecond range. Two laser systems, emitting at 1064 and 532 nm, were used. It was shown that the features obtained depend on whether the substrate is processed by means of pulse bursts or by grooves. In particular, when the samples were processed by grooves, machined depth, removed volume and ablation yields reached their maximum, placing the sample out of focus. It was shown that these characteristics do not depend on the processing conditions, the wavelength or the optical configuration, and that this is intrinsic behavior of the processing method. Furthermore, the existence of a close relation between material hardness and ablation yields was demonstrated.

## 1. Introduction

Laser processing is of great interest in the field of optics, electronics, microelectronics, aerospace and medicine. This technique is cost-effective compared to traditional methods and it may be applied to a wide range of substrates, such as metals, ceramics and semiconductors [1]. In the field of laser processing of materials, several methods such as laser machining, micro-machining, marking, drilling and pulsed laser deposition have been developed [2,3]. Furthermore, laser processing has been incorporated in industry in recent decades. Reductions in the costs of production, staff and maintenance and tool wearing make laser processing the most suitable working tool for machining hard and brittle materials such as advanced ceramics or glass-ceramic materials.

The discovery of techniques for generating short and ultra-short laser pulses, ranging from tens of nanoseconds to a few femtoseconds, has led to the development of more powerful systems, with power densities that can reach Terawatts/cm^2^. These laser systems, with better features and lower prices, offer a high-speed/high-quality tool for laser machining, which is of great interest in both basic and applied research for scientific and technological purposes [3,4]. Laser ablation depends on laser wavelength, optical features of laser beam, pulsewidth range, machining method and on the optical-thermal-mechanical properties of the substrate to be processed. Some theoretical descriptions have been developed by many authors to generalize the stages of the ablation process: laser radiation absorption, heat transfer to the target, evaporation and gas-dynamic of the vapor [5,6,7,8,9,10,11,12].

Laser machining of advanced ceramics has been developed over the last decades to achieve functional and structural components because of their outstanding features, such as their lightness, hardness, wear resistance and chemical stability at high temperature [13,14,15,16]. In particular, 8 mol % Yttria Stabilised Zirconia, 8YSZ, has commonly been used as an anode or electrolyte of solid oxide fuel cells in energy applications [17,18,19,20,21] and alumina as a bioceramic material, because of its biocompatibility, in scaffolds or implants [22,23].

Glass-ceramic materials have been commonly used as photonic materials for applications in optical amplifiers, tunable solid-state lasers, up-conversion luminescence devices, *etc* [24,25,26,27]. Glass-ceramic substrates present a great interest for industrial and engineering applications over other ceramic and glassy materials because of their good chemical inertness, high temperature stability and glass transition temperature, low coefficient of linear expansion, excellent thermal shock resistance and superior mechanical properties such as abrasion or impact. Furthermore, laser processing and surface modification of these materials is very interesting for functional purposes in industrial applications and these modifications may be applied as thermal-barriers, heat-conductor tracks, inclusion of thermal sensors, *etc* [28,29,30,31,32].

When a laser machining process is to be carried out, the first question which arises is where to place the sample with respect to the focal plane. At first glance, the answer could seem obvious: at the focal plane since this is the place where the beam waist takes its minimal value and therefore irradiance and fluence are maximal. However, as we showed in a previous work, the reference position and the machining method greatly influence the geometrical dimensions as well as the ablation yields obtained [33]. The main conclusion of this previous work was that when the samples were machined by grooves, the maximal ablation yield, machined depth and removed volume were reached by placing the sample out of focus, and not at focus as would be expected. Furthermore, we showed that there was a close relation among the ablation yields and the mechanical properties of the processed substrate.

The aim of this work is to demonstrate that in the nanosecond range, these characteristics are an intrinsic behavior of this machining method, and, hence, the maximal ablation yield, machined depth and removed volume are always reached by placing the sample out of focus. This apparently strange phenomenon is independent of the processing conditions in which it is carried out. Although the optical characteristics of the laser beam, optical configuration and processing conditions*—i.e.*, laser wavelength, optical lens, working frequency and the scanning speed—were modified, the phenomenon holds. The relevance of this result lies in the fact that in spite of any processing condition which may be modified, the optimal ablation results are obtained out of focus in this machining method. Furthermore, in this work, we demonstrate that the close relation between material hardness and ablation yields also holds independently of the processing conditions. This result is also of great importance since it demonstrates that laser machining can be used as a non-contact technique to determine material hardness. The main advantage of this technique would be that hardness can be measured not only at room temperature but also at high temperature ranges at which traditional measurement techniques cannot be used.

## 2. Experimental

### 2.1. Laser Processing

To study and compare the machining process under different wavelengths, two commercial laser systems were used:

A diode-pumped Q-Switch Nd:YAG laser system (E-line 20, Rofin-Sinar, Bergkirche, Germany). This system operates at its fundamental wavelength, 1064 nm, in Gaussian mode TEM_00_ with a beam quality factor M^2^ < 1.3 and a maximum mean power of 11 watts. An optical lens with focal length F of 100 mm placed at the output was used.

A diode-pumped Q-Switch Nd:YVO_4_ laser system (TruMark 6230, Trumpf, Grüsch, Switzerland). This system operates at a wavelength of 532 nm, Gaussian mode TEM_00_ with a beam quality factor *M*^2^ < 1.2 and a mean power of 7.2 watts. An optical lens with focal distance F of 320 mm was used.

Both laser systems are equipped with a programmable galvanometer at the output of the cavity controlled by Computer-Aided Design, CAD, software. In this way, the beam can be deflected making a bidirectional movement in such a way that any predefined pattern and processing procedure can be performed. The machining process is controlled by the diodes pump current *I_p_* (in relation to peak power), pulse frequency *f*, linear speed *V_L_* and distance between adjacent lines Δ. Taking into account the features of each laser system and using the equations [1]:

(1) $$D_{bw}=\frac{4F\;M^{2}\lambda }{\pi D_{0}}$$

(2) $$R=\left(\frac{\pi \;{D_{bw}}^{2}}{4\;M^{2}\lambda }\right)$$

where *D*_0_ is the diameter of the laser beam before the optical lens, the diameter at the focal point *D_bw_* and the Rayleigh range *R* are approximately 13 μm and 96 μm, respectively, for the Nd:YAG laser system and 37 μm and 1684 μm, respectively, for the Nd:YVO_4_ laser system.

Sheets of Ceran Suprema^®^ produced by Schott were used as glass-ceramic substrate. As ceramic substrates, dense alumina sheets with a content of 99% Al_2_O_3_ and dense substrates of zirconium oxide ceramic stabilized with 8% mol yttrium oxide, both produced by Kerafol, were used. The dimensions of the ceramic plates were 50 mm × 50 mm × 0.5 mm. In Table 1, thermal and mechanical properties of glass-ceramic and ceramic plates are presented.

**Figure 1:**
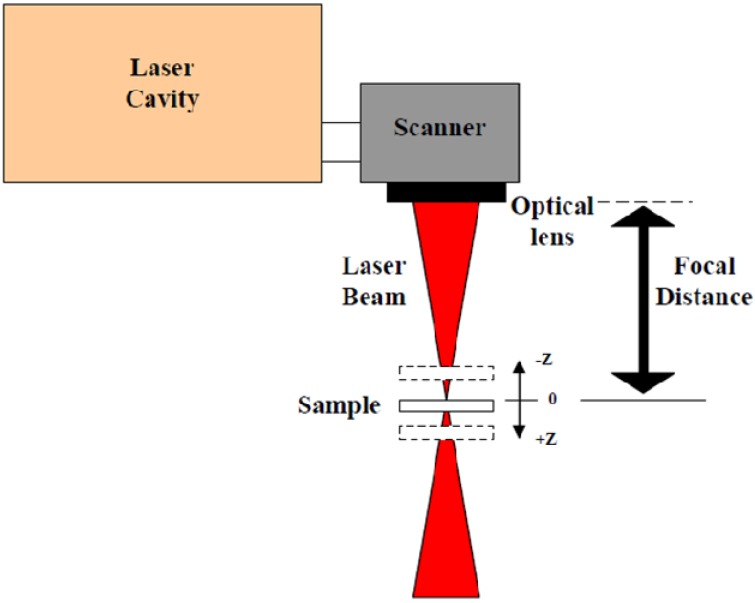
Sketch of the laser processing set-up.

Figure 1 shows a sketch of the laser processing. The sample is machined above and below the focal point, varying the reference position around it. The sign convention taken is: negative when the sample is moved upwards and positive when it is moved downwards. The laser processing was carried out by modifying the reference distance and using two methods: pulse bursts and machining grooves with dimensions 2 mm × 0.3 mm, length × width. Laser processing parameters are listed in Table 2.

**Table 1:** Properties of glass-ceramic and advanced ceramic substrates.

Property	Ceran Suprema	8YSZ	Al_2_O_3_
Density (g/cm^3^)	2.5 ^a^	5.85 ^b^	3.88 ^b^
Bending strength (MPa)	110 ^a^	265 ^b^	500 ^b^
Hardness Vickers	800	1200	1500
Thermal conductivity (W/mK)	1.7 ^a^	2.5 ^b^	25 ^b^
Thermal diffusivity (m^2^/s × 10^−6^) ^c^	0.85	1.07	7.58
Melting temperature (K)	1498 ^a^	2950 ^b^	2327 ^b^
Diffuse reflectance (1064 nm)	0.93	0.95	0.84
Diffuse reflectance (532 nm)	0.96	0.93	0.86
Optical absorption α |_1064 nm_ (cm^−1^)	3.52	9.34	10.90
Optical absorption α |_532 nm_ (cm^−1^)	28.37	40.72	50.27
Absorption length *L*_α_ = α^−1^|_1064 nm_ (cm)	0.28	0.11	0.09
Absorption length *L*_α_ = α^−1^|_532 nm_ (cm)	0.04	0.02	0.02
Thermal diffusion length *L*_th _|_10 ns_ (μm)	0.18	0.20	0.55

^a^ Schott Technical Data; ^b^ Kerafol Technical Data; ^c^ Measurement carried out at the Institute of Ceramic and Glass.

**Table 2:** Laser beam characteristics used to machine the substrates.

Wavelength (nm)	Frequency (kHz)	Pulse Energy (mJ)	Pulse-width (ns)	Peak Power (kW)
1064	1	2.7	8	300
1064	2	2.45	10	260
532	15	0.24	7.5	33.2

### 2.2. Characterization Techniques

Mechanical characterization was determined by a microhardness tester (Matsuzawa MXT-70, Tokio, Japan). Superficial topography and profile measurements have been carried out with an optical confocal microscope (Nikon Sensofar Plμ2300, Terrassa, Spain). Absorbance spectrum and diffuse reflectance were measured using a double beam spectrophotometer (UV-Vis-IR Cary 500 Varian, Santa Clara, CA, USA).

## 3. Results and Discussion

Ablation parameters such as ablation yield, machined depth and removed volume depend mainly on the amount of energy deposited on the substrate. This amount of energy irradiated over the substrate can be controlled taking into account the optical features of the laser beam, mainly the laser wavelength, which is in relation to the material absorption, the optical configuration of the laser system,* i.e.*, the focal distance of the optical lens since it is related to the area in which the laser energy is comprised, and finally with the processing method. When the whole laser pulses interact over the same area,* i.e.*, machining by pulse bursts, the laser processing is carried out by means of a percussion effect. When the laser beam is scanned,* i.e.*, machining by grooves, the laser pulses are overlapped by adjusting the processing parameters such as working frequency, scanning speed and distance between adjacent lines. As we showed in a previous work [33], there exists a great unexpected influence of the ablation parameters with the sample position with respect to the focal plane depending on the processing method.

**Figure 2:**
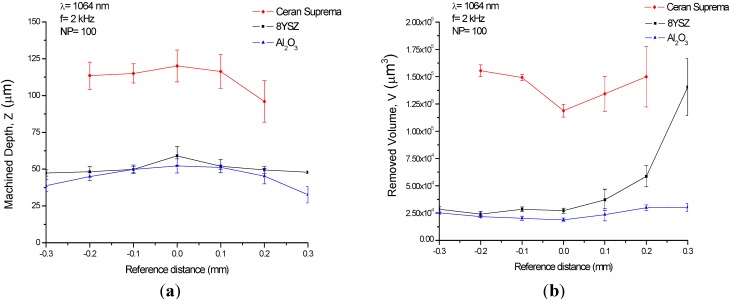
(**a**) Machined depth, *Z*; and (**b**) removed volume, *V*, obtained machining the substrates by means of 100 laser pulses of 2.45 mJ at a wavelength of 1064 nm with pulse-width of 10 ns and a working frequency of 2 kHz.

When the substrate is machined by using pulse bursts, we showed that the machined depth and the removed volume were maximal and minimal respectively at the focal point. As the surface to be processed was moved upwards or downwards from the focal plane, the machined depth decreased and the removed volume increased. As an example, Figure 2 shows the machined depth and the removed volume obtained when 8YSZ, Al_2_O_3_ and glass-ceramic sheets were machined by means of pulse bursts using 100 laser pulses of 2.45 mJ at a wavelength of 1064 nm with pulse-width of 10 ns and a working frequency of 2 kHz. The values of the machined depth obtained at the focal point were 0.52, 0.59 and 1.20 μm/pulse for Al_2_O_3_, 8YSZ and the glass-ceramic substrate, respectively. With regards to the removed volume, values of 188 μm^3^/pulse for Al_2_O_3_, 272 μm^3^/pulse for 8YSZ and 1188 μm^3^/pulse for the glass-ceramic were recorded. Taking into account the pulse energy and discounting losses due to diffuse reflection, the ablation yields were 2.08, 0.45 and 0.14 MJ/cm^3^ for Al_2_O_3_, 8YSZ and glass-ceramic, respectively.

**Figure 3:**
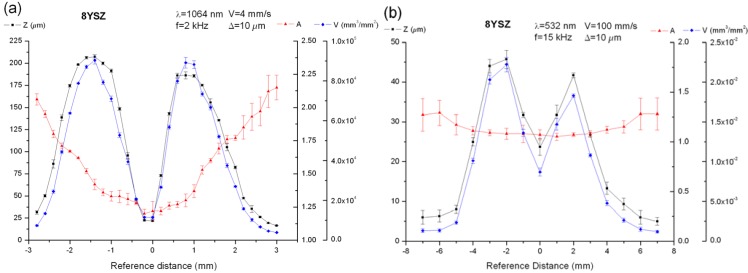
Geometrical dimensions obtained for 8YSZ substrates processed by means of grooves, machining at a wavelength of 1064 nm (**a**); and 532 nm (**b**).

However, when substrates were machined by groves, the behavior was completely different. In this machining method, ablated depth and removed volume were minimal in the vicinity of the focal point, reaching their maximum value when the sample was placed out of focus, as shown in Figure 3a, where the width, the machined depth and the removed volume are depicted for a 8YSZ substrate which has been processed at a wavelength of 1064 nm with a laser pulse energy of 2.45 mJ and pulse-width of 10 ns, using a repetition frequency of 2 kHz, an optical lens of 100 mm and a scanning speed of 4 mm/s with a distance between adjacent lines of 10 μm. The machined depth has been expressed in percentage scale, referred to 0.3 mm. The features of the sample allowed it to be machined up to 3 mm from the focal plane. Furthermore, although the maximal depth and removed volume were reached out of focus, the highest values were obtained when the sample was moved upwards and the focal point was placed in the inside. This asymmetry is related to the pressure exerted inside the substrate when the focus is placed in the inside which leads to a higher removed volume and machined depth.

**Figure 4:**
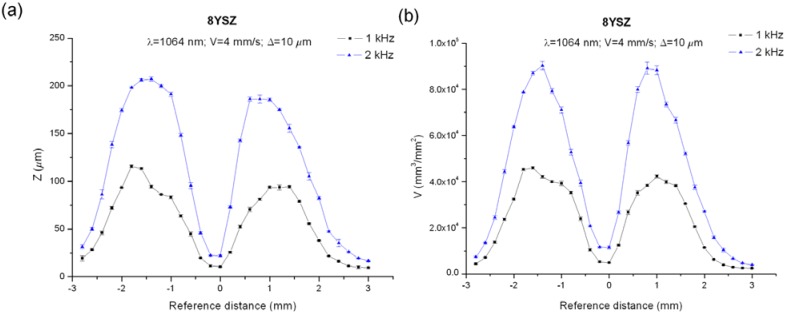
Machined depth, *Z*, (**a**) and removed volume, *V*; (**b**) obtained for 8YSZ substrates machining by grooves at a wavelength of 1064 nm with working frequencies of 1 and 2 kHz.

**Figure 5:**
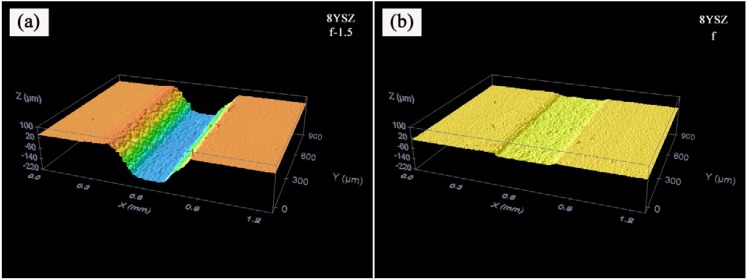
Topographies and profiles for 8YSZ substrates machined by grooves, −1.5 mm out of focus (**a**); and at focus (**b**).

In order to assess if this behavior is characteristic of this machining method, the 8YSZ samples have been processed by modifying the laser features, the optical configuration and the processing parameters in order to ascertain the dependence of this phenomenon to the parameters which control the irradiated energy over the substrate during the process,* i.e.*, material absorption, beam diameter and pulse overlapping. Firstly, maintaining the wavelength, the scanning speed and the distance between adjacent lines, the working frequency was modified processing the sample with 1 kHz so that the distance between adjacent pulses, given by the quotient *V_L_*/*f*, was doubled from 2 to 4 μm, thus reducing the energy irradiated over the interaction area from 6.13 to 3.38 J/mm^2^. Figure 4 shows the machined depth (a) and the removed volume (b) for both conditions. It can be observed that depth and volume followed the same tendency for both frequencies and their values were nearly doubled when the working frequency increased from 1 to 2 kHz. In particular, at focus for 1 and 2 kHz, the depth and the volume were 10.4 μm and 4786 mm^3^/mm^2^ and 22 μm and 11,374 mm^3^/mm^2^, respectively. The maximal values were obtained placing the focus around 1.5 mm inside the sample and were 115.6 μm and 45,901 mm^3^/mm^2^ and 207 μm and 90,261.5 mm^3^/mm^2^ for 1 and 2 kHz, respectively. As an example, Figure 5 shows the topographies and the profiles of the sample machined at focus (a) and −1.5 mm out of focus (b) for a working frequency of 2 kHz.

Next, the laser wavelength, the optical configuration and the working parameters were modified. The 8YSZ samples were processed by means of a Q-Switch Nd:YVO_4_ laser system emitting at 532 nm, using an optical lens of 320 mm and laser pulses of 0.24 mJ with a pulse-width of 7.5 ns, a repetition frequency of 15 kHz, and scanning the laser beam at 100 mm/s with a distance between adjacent lines of 10 μm. As Figure 3b shows, machined depth and removed volume followed the same behavior as previously reported for a wavelength of 1064 nm,* i.e.*, minimal depth and volume at focus and maximal values placing the sample out of focus with the highest values with the focus placed on the inside, in this case around 2 mm. The values obtained at focus and at the maximal point were 23.7 μm and 8.7 × 10^−3^ mm^3^/mm^2^ and 45 μm and 2.22 × 10^−2^ mm^3^/mm^2^, respectively. It is worth mentioning the fact that with this laser wavelength, the features of the substrate allowed it to be machined up to 7 mm beyond the focus plane. Although the optical absorption is higher for 532 nm than for 1064 nm, the processing conditions, with a higher distance between adjacent pulses *V_L_*/*f* of 6.6 μm, a lower pulse energy and peak power, led to lower values of machined depth and removed volume if compared with the ones obtained for 1064 nm. Just as an example, at focus, fluence and irradiance were 2034 J/cm^2^ and 226 GW/cm^2^ at 1064 nm for 1 kHz and 22 J/cm^2^ and 3.1 GW/cm^2^ at 532 for 15 kHz, around two orders of magnitude lower.

Therefore, the fact that the maximal ablation parameters are reached by placing the sample out of focus is independent of the processing conditions in which the processing is carried out means that phenomenon holds in spite of modification of the laser wavelength, focal length and processing parameters.

Laser ablation in the nanosecond range is a photothermal-mechanical process so that the material is removed by the thermal mechanisms activated by the laser beam. They essentially consist of the absorption of the laser energy and the subsequent evaporation and ejection of material involving a heat conduction transfer to the surrounding zones. Thus, a heat affected zone, HAZ, where composition, microstructure and mechanical properties of the substrate may be modified is produced [2]. The dimensions of the HAZ observed in the vicinity of the machined areas varied with the reference distance [33]. Micrographs and Energy Dispersive X-Ray Spectroscopy, EDX, analysis carried out over these areas showed no modifications in microstructure and composition. Likewise, hardness tests carried out on these areas showed that hardness remained almost unchanged, at approximately 1200 ± 100 HV.

**Figure 6:**
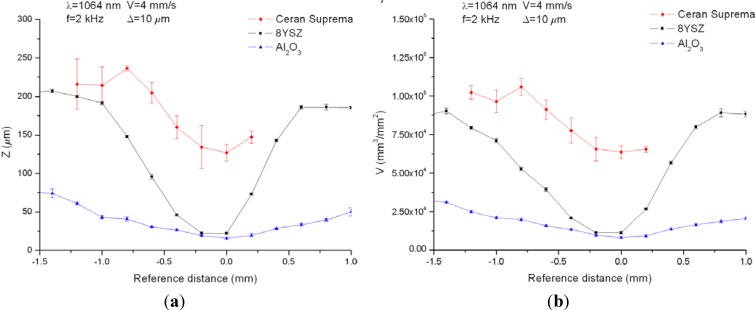
Machined depth (**a**) and removed volume (**b**) for 8YSZ, Al_2_O_3_ and glass-ceramic substrates processed at 1064 nm using a working frequency of 2 kHz, a scanning speed of 4 mm/s and a distance between adjacent lines of 10 μm.

Finally, the close relation found among hardness and ablation yields when the substrates were machined at a wavelength of 1064 nm was assessed for 532 nm. When 8YSZ, Al_2_O_3_ and glass-ceramic substrates were machined with the same working parameters at a wavelength of 1064 nm, an inverse relation was found between the material hardness and the laser processing results so that the harder the substrate the lower the machined depth and removed volume obtained. As an example, Figure 6 shows the machined depth (a), and the removed volume (b) for the three substrates processed using a working frequency of 2 kHz, a scanning speed of 4 mm/s and a distance between adjacent lines of 10 μm. In particular, at the focal point, 127 μm in depth with a removed volume of 6.36 × 10^4^ mm^3^ per square millimeter processed was obtained for the glass-ceramic substrate, whereas for 8YSZ and Al_2_O_3_, depths of 22 μm and 16 μm with removed volumes of 1.14 × 10^4 ^mm^3^/mm^2^ and 8.19 × 10^3 ^mm^3^/mm^2^ were obtained respectively. The energy per ablated volume, calculated taking into account the removed volume and the energy delivered per square millimeter, resulted in 0.13 J/cm^3^, 0.54 J/cm^3^ and 2.39 J/cm^3^ for glass-ceramic, 8YSZ and Al_2_O_3_ respectively,* i.e.*, the greater the yield the higher the hardness.

**Figure 7:**
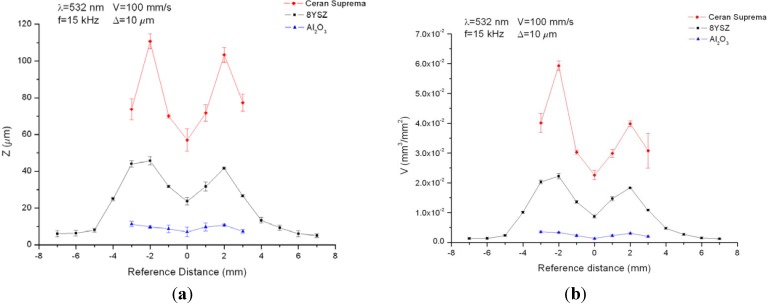
Machined depth (**a**) and removed volume (**b**) for 8YSZ, Al_2_O_3_ and glass-ceramic substrates processed at 532 nm using a working frequency of 15 kHz, a scanning speed of 100 mm/s and a distance between adjacent lines of 10 μm.

In order to compare the results obtained for the three substrates at 532 nm, Al_2_O_3_ and glass-ceramic substrate were processed with the same working conditions 8YSZ was processed with, as shown in Figure 7a,b. In this case, the behavior obtained was the same than for the NIR case but the differences were much greater. At focus, the machined depth and the removed volume were 7 μm and 1.27 × 10^−3^ mm^3^/mm^2^, 24 μm and 8.7 × 10^−3^ mm^3^/mm^2^ and 57 μm and 2.26 × 10^−2^ mm^3^/mm^2^ for the Al_2_O_3_, 8YSZ and the glass-ceramic, respectively, which gave rise to the following ablation yields: 396.87 J/mm^3^ for Al_2_O_3_, 28.96 J/mm^3^ for 8YSZ and 6.37 J/mm^3^ for glass-ceramic substrate.

## 4. Conclusions

8YSZ, alumina and glass-ceramic samples have been processed by laser ablation irradiating with pulse bursts and machining grooves in the nanosecond range, modifying the laser wavelength, the optical configuration and the processing parameters. The different behavior obtained depending on whether the processing technique utilized has been confirmed. Machining by grooves,* i.e.*, scanning the laser beam, shows an intrinsic behavior that is independent of the laser beam features as well as of the optical configuration or processing conditions. This behavior, which leads to minimal machined depth and removed volume at focus, and maximal values achieved placing the sample out of focus with the highest values achieved with the focus placed on the inside, is completely different than the one obtained machining by means of laser pulse bursts, in which the entire laser pulses interact over the same substrate, and the maximal machined depth is reached at focus.

The close relation between mechanical properties and ablation yields has been demonstrated. This relation is independent of the laser beam features, optical configuration or processing conditions in the nanosecond range. The higher the material hardness, the lower the machined depth and removed volume obtained. Furthermore, for lower wavelengths, the difference in the ablation results increases.
